# Teeth Lesion Detection Using Deep Learning and the Internet of Things Post-COVID-19

**DOI:** 10.3390/s23156837

**Published:** 2023-07-31

**Authors:** Imran Shafi, Muhammad Sajad, Anum Fatima, Daniel Gavilanes Aray, Vivían Lipari, Isabel de la Torre Diez, Imran Ashraf

**Affiliations:** 1 College of Electrical and Mechanical Engineering, National University of Sciences and Technology (NUST), Islamabad 44000, Pakistan; imranshafi@ceme.nust.edu.pk (I.S.); anumfatima427@gmail.com (A.F.); 2 Abasyn University Islamabad Campus, Islamabad 44000, Pakistan; muhammad_sajad47@yahoo.com; 3 Higher Polytechnic School, Universidad Europea del Atlántico, Isabel Torres 21, 39011 Santander, Spain; daniel.gavilanes@uneatlantico.es (D.G.A.); vivian.lipari@uneatlantico.es (V.L.); 4 Universidad Internacional Iberoamericana, Campeche 24560, Mexico; 5 Fundación Universitaria Internacional de Colombia Bogotá, Bogotá 11131, Colombia; 6 Universidad Internacional Iberoamericana Arecibo, Puerto Rico, PR 00613, USA; 7 Universidade Internacional do Cuanza, Cuito EN250, Bié, Angola; 8 Department of Signal Theory, Communications and Telematics Engineering, Unviersity of Valladolid, Paseo de Belén, 15, 47011 Valladolid, Spain; 9 Department of Information and Communication Engineering, Yeungnam University, Gyeongsan 38541, Republic of Korea

**Keywords:** teeth lesion detection, IoT enabled framework, transfer learning, automated detection model, AlexNet

## Abstract

With a view of the post-COVID-19 world and probable future pandemics, this paper presents an Internet of Things (IoT)-based automated healthcare diagnosis model that employs a mixed approach using data augmentation, transfer learning, and deep learning techniques and does not require physical interaction between the patient and physician. Through a user-friendly graphic user interface and availability of suitable computing power on smart devices, the embedded artificial intelligence allows the proposed model to be effectively used by a layperson without the need for a dental expert by indicating any issues with the teeth and subsequent treatment options. The proposed method involves multiple processes, including data acquisition using IoT devices, data preprocessing, deep learning-based feature extraction, and classification through an unsupervised neural network. The dataset contains multiple periapical X-rays of five different types of lesions obtained through an IoT device mounted within the mouth guard. A pretrained AlexNet, a fast GPU implementation of a convolutional neural network (CNN), is fine-tuned using data augmentation and transfer learning and employed to extract the suitable feature set. The data augmentation avoids overtraining, whereas accuracy is improved by transfer learning. Later, support vector machine (SVM) and the K-nearest neighbors (KNN) classifiers are trained for lesion classification. It was found that the proposed automated model based on the AlexNet extraction mechanism followed by the SVM classifier achieved an accuracy of 98%, showing the effectiveness of the presented approach.

## 1. Introduction

Dental diseases such as periodontal disease, dental caries, and fluorosis affect a large population worldwide. However, their prevalence varies among different geographical distributions. Based on the research conducted by World Health Organization, dental diseases affect 621 million children, and almost 2.40 billion adults are affected by dental caries [1]. Furthermore, results obtained from the National Health and nutrition examination survey show that almost 41% of children between the age of 2–11, 42% of adolescents from 6–19 years of age, and 90% of adults over the age of 20 are affected by dental caries [2].

Periapical radiographs, a kind of intraoral radiograph, are widely used in endodontics. Periapical diseases are substantially inflammatory lesions that lead to dental caries causing detrimental injury to the teeth. These diseases are classified as apical cysts, abscesses, and granulomas affecting the dental pulp protected by enamel, cementum, and dentin. If left untreated, chronic damage to the pulp chamber can lead to inflammation, eventually turning into pulp necrosis and periradicular pathosis. Scenarios triggering periapical radiolucency include trauma, tooth wear, or caries. However, it is possible to prevent their spread through non-surgical endodontic treatment. Most periapical lesions heal through meticulous nonsurgical endodontic treatments. A timeframe of 6 to 12 months is required for assessing the healing potential, after which root canal treatment should be considered [3,4]. Additionally, complete healing of the lesion might take up to four years if not diagnosed in time. This requires frequent visits and interactions with the dentist and associated staff, which has become difficult in the post-COVID-19 environment. Postponing treatment can increase the risk of tooth fracture [5]. Therefore, it is essential to diagnose teeth lesions in a timely way through routine oral examination of the teeth and gums as well as the soft tissues in and around the mouth. Conventional periapical radiographs are obtained by exposing X-ray radiation that is then processed chemically to produce images. This film-based conventional method has certain drawbacks, and digital radiography has been introduced to overcome these drawbacks. This process involves acquiring images digitally which are then manipulated using a computer. There are several ways of obtaining such images, including intraoral sensors, charge-coupled devices, and scanning of radiographs [6]. Due to the emergence and spread of low-cost connectivity in underdeveloped and developing countries, the Internet of Things (IoT) has enabled dental services to be more broadly leveraged, providing better dental health in developing countries and decreasing the overall demand for dental care. Looking at the successful adoption of the IoT in different sectors [7,8], it can serve as an attractive area of adoption in dentistry. IoT-enabled devices can obtain information, including pathological parameters, to predict oral health and make decisions regarding the treatment of the disease at earlier stages. With the growth in IoT-based healthcare services, it has become possible to achieve significant enhancement in dental healthcare, including early detection and prediction [9].

Recent advancements in the microcontroller-based wearable and implantable smart electronic devices has given rise to touchless technologies, which are gaining momentum and are regarded as the future of medical science. Such wearables are data transmission devices worn on the human body, such as the eyes, ears, knees, feet, fingers, and hands. These devices can detect, process, analyze, receive, and transmit related vital body signals and information such as the pulse rate, heart rate, body temperature, and other ambient data which do not require human intervention and may allow immediate biofeedback to the wearer [10,11]. They are an essential part of an IoT scenario in which they allow the exchange of high-quality data through the global internet via processor and relay agents to the operator, data collector, company, and/or manufacturer.

**Figure 1:**
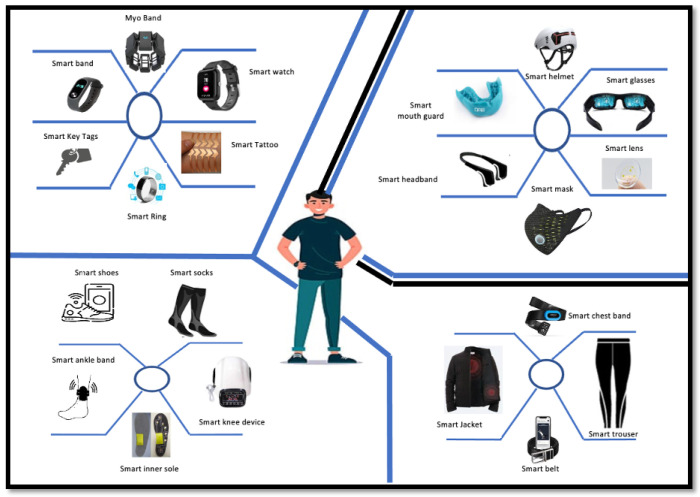
Wearable healthcare devices.

As a result, effective biomedical monitoring systems can be designed for medical diagnosis, physiological and psychological health monitoring, and evaluation through continuous measurement of critical biomarkers from various human body parts. A possible range of wearable devices in the form of basic clothing, common accessories, attachments, and body implants is shown in Figure 1.

**Figure 2:**
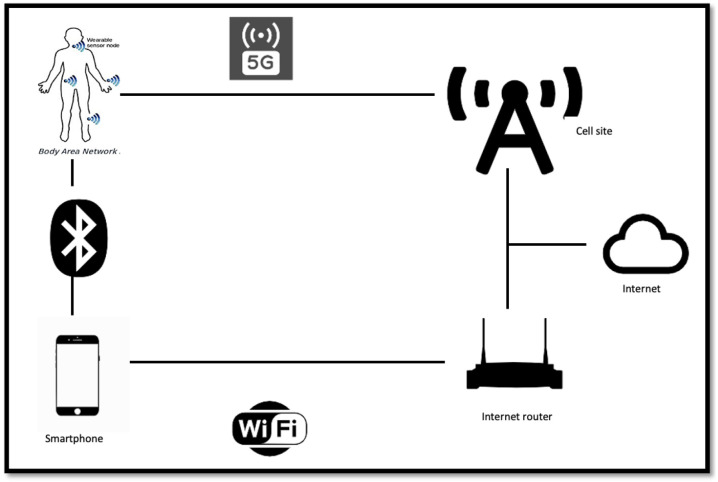
IoT communication infrastructure.

The information available from these sensors can be transmitted through modern communication links, as shown in Figure 2, for intelligent processing and storage in an IoT environment. The IoT infrastructure makes possible the monitoring and recording of real-time temporal characteristics of chronic/acute patients, allowing for both immediate diagnosis and further research around finding and tracking disease trends.

Wearable technology is expanding into newer applications, and has gained significant space in consumer electronics through smartwatches, activity trackers, advanced e-textiles, healthcare products, navigation systems, and geofencing applications. Within the healthcare domain, parameters such as blood pressure, exercise time, steps walked/ran, running speed, calories burned, heart rate, body temperature, pulse rate, seizures, physical stress/strain, and level of certain body chemicals can be measured and used to evaluate users’ health. The issue of power sourcing for wearables has been suitably addressed by various methods as well. Smart wristbands can be used to generate their own electrical signals through thermoelectric generation technology (TEG) [12]. Power can be generated using the temperature differential between human skin and the environmental temperature. These implants have been used to monitor the diet and nutrition content of meals, and play a great role in improving diet plans, leading to a more healthy and fit life [13]. These devices collect information about salt, glucose, and even alcohol consumption, can share these data with other smart devices that a person uses, and are capable of updating physicians about diet routines and health conditions.

Additionally, IoT infrastructure allows monitoring and recording of the real-time temporal characteristics of chronic/acute patients for immediate diagnosis and tracing of disease trends. For diagnosing dental caries, digital radiographs are employed by dentists to assess and evaluate the extent of caries and to determine the need for treatment. Among the common intraoral types of radiographs (periapical, bitewing, and panoramic), periapical radiographs are the more common, as they provide localized information related to the length and adequacy of caries and periodontal ligament space [14]. Furthermore, challenges faced in interpreting conventional radiographs create the potential for inconsistencies among dentists due to a number of factors, including contrast variation, magnification, and lack of experience. Nevertheless, due to the clinical reliability of periapical radiographs these have become a popular choice for diagnosing dental caries. Additionally, the above-mentioned challenges pave the way for utilizing automated solutions for improving diagnosis and standardization of care.

Recently, deep learning-based techniques have demonstrated excellent performance in computer vision, including object detection, tracking, and recognition, improving the ability to build software for automated analysis and evaluation of images. Different deep learning techniques have demonstrated the potential for automated identification of radiological and pathological features. Furthermore, different image processing and recognition procedures have been adopted for medical segmentation, with high accuracy and efficiency observed in the classification of different diseases using deep learning-based models, including cystic lesions [15], skin cancer [16], COVID-19 [17], and thoracic diseases [18], demonstrating improved accuracy and efficiency.

On the contrary, few studies have been observed based on deep learning, specifically deep convolutional neural networks (CNNs), in the dental field, limiting research investigating the diagnosis and detection of dental caries. Additionally, limited attempts at automatic dental radiograph analysis using deep learning have been observed from previous studies [19,20,21]. Other than this, variable accuracy is observed in the methods currently utilized, highlighting the need for further research in this field. Approaches involving CNN and transfer learning have been used in dental diseases based on dental X-rays [22,23].

However, due to ease of classification, most approaches consider specific issues such as periodontitis, periapical lesions, and dental caries. Other teeth lesions, such as endo-perio and perio-endo lesions, are more challenging to diagnose due to their closely matching attributes. Therefore, they have been grouped into five significant lesions: primary endodontic with or without secondary periodontal involvement, primary periodontal with or without secondary endodontic involvement, and true combined lesion [24,25].

There are several limitations and challenges in the existing approaches. First, most of the work relies on traditional machine learning techniques, which may not be effective in detecting complex lesions due to the closely matching attributes of dental lesions, making them difficult to diagnose using traditional methods. Second, data insufficiency is a common challenge, as there may not be enough data available to train accurate models.

Keeping in view the limitations of previous studies, the present study aims to predict five types of endo-perio and perio-endo lesions in periapical radiographs by adopting a hybrid approach using transfer learning and a fine-tuning method after acquiring radiographs from a wearable device through an IoT infrastructure. Additionally, the presented work compares the diagnosis efficacy of different machine learning-based techniques, including support vector machine (SVM) and K-nearest neighbors (KNN), on features extracted using a pretrained AlexNet. Finally, the objective is to evaluate the efficacy of CNN algorithms and transfer learning to detect and classify dental caries in periapical radiographs and to overcome data insufficiency using data augmentation techniques. The main contributions of this research include:

- Effective detection and classification of dental caries in periapical radiographs by leveraging deep learning techniques such as transfer learning and image augmentation;
- Integrating IoT technology through an IoT-enabled device to capture tooth lesions from radiographs, allowing for remote monitoring and management of dental health, which is significant in situations where in-person visits to dental clinics might be limited.

The rest of the paper is structured in five sections, with Section 2 presenting a literature review on wearables in the healthcare sector and machine, deep, and transfer learning concepts and techniques used for diagnosis of teeth lesions in dental X-rays, Section 3 explaining the methodology of the proposed framework, Section 4 discussing the results and providing a comparative analysis, and Section 5 concluding the paper.

## 2. Related Work

Recently, new technologies have been evolving rapidly, and the need for these technologies has increased due to the COVID-19 pandemic. The IoT, specifically the Internet of Dental Things (IoDT), is a novel strategy that aids in managing and preventing dental caries as well as periodontal and other disorders [9,26]. Diagnostic screening and visualization incur significant costs, as dental diseases are very common. With the development of the Internet of Things (IoT), internet-based systems have shown great potential in healthcare, particularly dental healthcare. Smart dental IoT-based systems based on intelligent hardware and deep learning can allow for better identification and monitoring, improving the preventive care process.

Over the past decade, various retention and restoration methods have been proposed for detecting dental caries. However, there remains room for improvement in diagnosing dental caries due to various teeth morphologies and restoration shapes [27]. Furthermore, detecting lesions in their earlier stages is challenging using these methods. The final diagnosis ultimately depends on empirical evidence, even though different dental radiographs (periapical, panoramic, and bitewing) are widely used. A range of options are available to detect dental lesions, including real-time ultrasound imaging [28], contrast media, Papanicolaou smears, and cone beam computed tomography (CBCT), with the latter showing the highest discriminatory ability. However, it is limited in general dental practice due to its cost and high radiation dose [29]. Panoramic radiographs allow all teeth to be assessed simultaneously; however, these methods have proven to be less accurate due to limited datasets. Therefore, using other imaging systems, such as periapical radiographs, which is the current standard of endodontic radiography, can enhance the chance of more accurate preoperative diagnosis.

Regardless of their discriminatory ability, the reliability of the radiographs depends on different factors, including the examiner’s reliability and experience. To overcome this, automated systems for dental radiographs using machine learning and deep learning techniques have demonstrated high performance. Patil and fellow authors [30] proposed an attractive model for caries detection that detects dental cavities using MPCA-based feature extraction and an NN classifier trained using an adaptive DA algorithm to classify extracted features. Nonlinear programming optimization is used to maximize the distance between the feature output. This approach performs better than methods such as PCA, LDA, MPCA, and ICA, reaching 95% accuracy on three developed test cases. In addition, the proposed model works better than machine learning techniques such as KNN, SVM, and Naıve Bayes for dental lesion detection.

Kühnisch et al. proposed a deep learning approach using a convolutional neural network (CNN) trained using image augmentation and transfer learning to detect, categorize, and compare the diagnostic performance. The dataset comprised 2417 anonymized photographs of permanent teeth with 1317 occlusal and 1100 smooth surfaces. The images were organized into three main categorie: non-cavitated caries lesion, caries-free, and caries-related cavitation. The proposed model was able to classify caries in 92.5% of all images and 93.3% of all tooth surfaces, achieving an accuracy of 90.6% for caries-free surfaces, 85.2% for non-cavitated caries lesions, and 79.5% for cavitated caries lesions [19].

Privado et al. [31] reviewed different studies investigating caries detection using neural networks with different dental images. Various neural networks for detection and diagnosis of dental caries were discussed, and a database was constructed containing each neural network’s parameters. In 2019, Casalegno et al. [32] presented a deep learning model for the automatic detection and localization of dental lesions in near-infrared TI images based on a convolutional neural network (CNN). Their dataset consisted of 217 grayscale images of upper and lower molars and premolars obtained using the DIAGNOcam system. Over 185 training samples were used to train the model. The model’s performance was tested based on pixel segmentation into classes and binary labeling of the region of interest. In addition, the model was tested using Monte Carlo cross-validation. However, the proposed model showed physically unrealistic labeling artifacts, especially in underexposed and overexposed regions. Geetha et al. [33] proposed a dental lesion classification model based on an Artificial Neural Network (ANN) and performed ten-fold cross-validation, achieving an accuracy of 97.1%.

Duong et al. formulated a computational algorithm to automate and recognize carious lesions on tooth occlusal surfaces using smartphone images. The images were evaluated for caries diagnosis using International Caries Detection and Assessment System (ICDAS) II codes. Support vector machine (SVM) was used for classification; the proposed model was able to diagnose caries with a highest accuracy of 92.37%. Schwendicke and Paris discussed the cost-effectiveness of using artificial intelligence in caries detection. It was found that in most cases using AI is more effective and cost-efficient [34]. Gracia Cantu proposes a model involving training a CNN; the model was validated and tested on 3686 collected bitewing radiographs. However, this research involved proximal caries lesions on permanent teeth. To measure cost-effectiveness, a Markov simulation model was used. Several studies have used AI, more specifically, deep learning techniques, to analyze dental images. These studies have mainly focused on developing and evaluating models that are more accurate and efficient [20].

A deep convolutional neural network to detect caries lesions in near-infrared light transillumination images was proposed by Schwendicke and Rossi. The model involves two CNNs, namely, Resnet 18 and Resnext50, which were pre-trained on the ImageNet dataset. The dataset was processed digitally before being fed into the CNN. Ten-fold cross-validation was used to validate the model, which was optimized with respect to recall, F1 score, and precision. The model obtained an accuracy of 0.73 (0.67/0.80) using Resnet18 and an accuracy of 0.74 (0.66/0.82) using Resnext50, with a mean 95% confidence interval (CI). However, there are certain limitations of deep neural networks. One is that they are opaque prediction models and have a complex and nonlinear structure that makes it difficult to make decisions based on their results. Furthermore, limited augmentation and optimization processes are performed, and higher accuracies can be achieved by combining this approach with a larger dataset and in different settings with different diagnostic standards [21].

Another interesting model was formulated by Saleh et al. [35], combining a deep convolutional neural network (CNN) and optical coherence tomography (OCT) imaging modality to classify human oral tissues for earlier detection of dental caries. The CNN utilized two convolutional and pooling layers for feature extraction, and used the probabilities of the SoftMax classification layer to classify each patch. The sensitivity and specificity for oral tissues were found to be 98% and 100%, respectively. Thus, this model can help to classify oral tissues with various densities for earlier dental caries detection [10].

Prajapati and Nagaraj [22] proposed a model that combines a deep convolutional neural network (CNN) and optical coherence tomography (OCT) imaging modality to classify human oral tissues for earlier detection of dental caries. Their CNN utilized two convolutional and pooling layers for feature extraction and used the probabilities of the SoftMax classification layer to classify each patch. The sensitivity and specificity for the oral tissues were found to be 98% and 100%, respectively. Thus, this model can help to classify oral tissues with various densities for earlier dental caries detection. An automatic lesion detection model to analyze and locate lesions in panoramic radiographs using different image processing techniques was proposed by Bridal et al. in [36]. Their system was capable of root localization, tooth segmentation, jaw separation, and detection of periapical lesions. First, the input image is enhanced by observing the smooth variations between intensities of neighboring pixels, then a Gaussian filter is used to smooth the photos. The jaws are separated by feeding discrete wavelet transformation into polynomial regression, then tooth segmentation and apex localization are performed. Their model achieved a specificity and specificity of 89% and 70%, respectively.

Ghaedi et al. [37] proposed a method for examining dental caries in which dental caries are examined using optical images and the histogram equalization method. Segmentation is achieved in two steps; first, the circular Hough transform and region growth is used to the segment the tooth surface. Second, the morphology method is applied to identify unstable regions within the tooth boundaries; the authors extracted 77 features from these unstable regions when using a suitable window size. Feature space is reduced using a heuristic approach based on the information gain ratio method. Gawad et al. [38] formulated a caries status detection and classification model based on a low-powered and less hazardous 635 nm He-Ne laser–tissue interaction mechanism to characterize human teeth into regular, moderate, and severe caries degree status.

Similarly, another study provided a framework for diagnosing periodontal, periapical, and dental caries using the CNN and transfer learning approaches [23]. This method employed a CNN consisting of five convolution layers, four fully connected layers, and two max-pooling layers. The transfer learning technique was used in two different ways. First, a pre-trained VGG16 (a CNN model used for classification and detection) was combined with another CNN trained earlier. Eight convolutions, four zero padding, five max pooling, and eight fully connected layers were used. Second, the pretrained VGG16 model was fine-tuned using a dataset and the results were analyzed based on accuracy.

Table 1 summarizes the related research performed by different authors for the detection of teeth lesions using deep learning approaches. Although several studies have incorporated artificial intelligence, and more specifically deep learning techniques, for dental lesion detection, there remains room for further improvement in analyzing and classifying dental caries.

## 3. Methods and Materials

**Figure 3:**
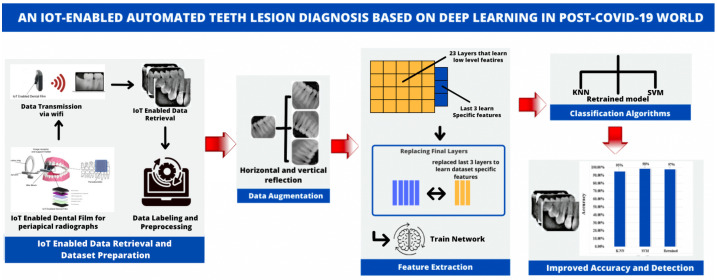
The architecture of the proposed framework.

Figure 3 illustrates the proposed system for automatic dental lesion detection. The automatic classification of teeth lesions is divided into five steps: (1) IoT-enabled data retrieval and dataset preparation (2) data augmentation; (3) feature extraction; (4) classification; and (5) improved accuracy and detection.

### 3.1. Materials

#### 3.1.1. IoT to Enable Data Retrieval

**Figure 4:**
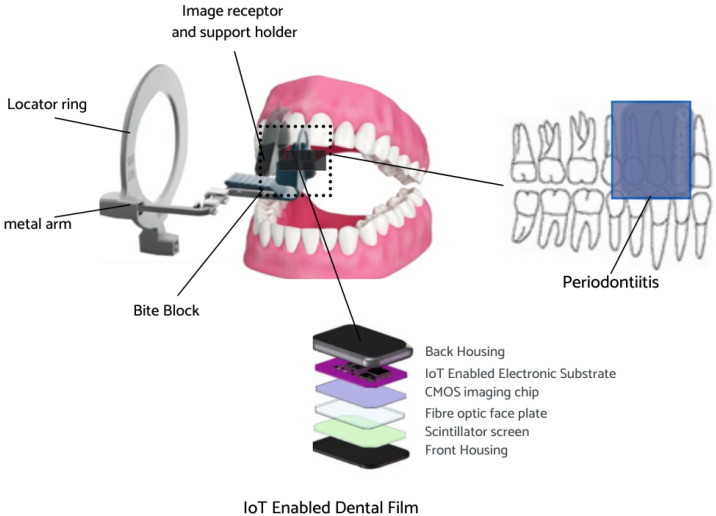
IoT-enabled dental film placement for periapical radiographs.

The Internet of things (IoT) is a collection of physical objects which enable collection and exchange of information over the network. IoT devices allow gathering real-time data from sensors to allow better decision-making [42]. The IoT-enabled dental film utilized in the present work involves a circuit board with a proximity sensor to ensure that the film is placed securely on the patient’s teeth, another sensor to measure the rotation of the head in order to acquire a proper angle, and a WiFi module for data transmission. The IoT-enabled dental film is placed on the extension cone parallel (XCP) device to take periapical radiographs of the posterior teeth [43]. The placement for periapical radiographs and the regions captured is illustrated below in Figure 4. The face of the dental film is positioned perpendicular to the interdental space between the teeth. The film is guided towards the midline, the patient bites into the bite block, and then the X-ray is taken [44].

**Figure 5:**
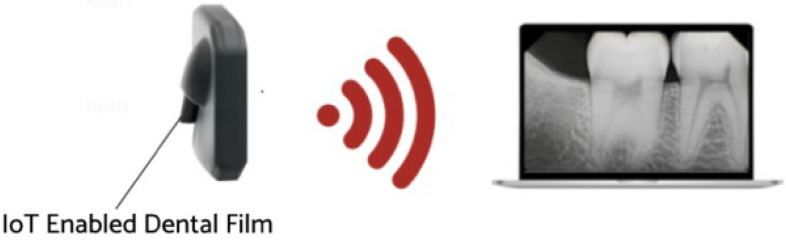
Wireless radiograph transmission.

After acquiring the radiographs, the WiFi module (ESP8266) mounted inside the circuit transmits the images automatically to the laptop/smartphone for further analysis. This module is specifically designed for IoT for wireless connectivity [45]. The proposed system can help to reduce the proximal surface overlap in periapical radiography [46]. Experimentation was carried out at a dental institute in Rawalpindi, Pakistan, and 534 periapical radiographs labeled by experienced clinicians were collected. Figure 5 below depicts the process of wireless radiograph transmission from IoT-enabled dental film to a laptop/tablet/smartphone screen or any smart device through WiFi.

#### 3.1.2. Data Preparation

**Figure 6:**
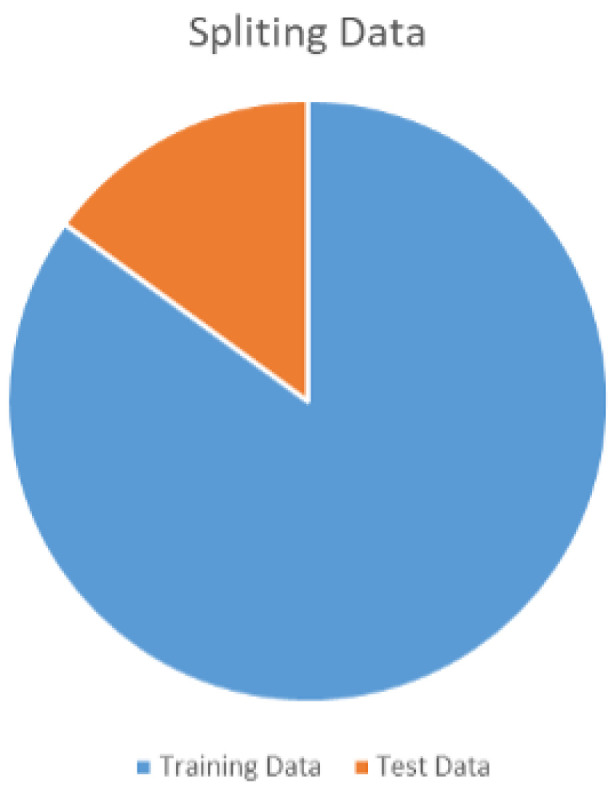
Traing and test dataset ratio.

The periapical radiograph dataset is divided into two sets for training and testing, containing 453 and 81 images, respectively. The data division ratio is shown below in Figure 6.

Radiographs captured and transmitted through IoT-enabled devices were labeled for their classes by experts. The class distribution for the training and test datasets is shown in Table 2.

### 3.2. Methods

#### 3.2.1. Data Augmentation

**Figure 7:**
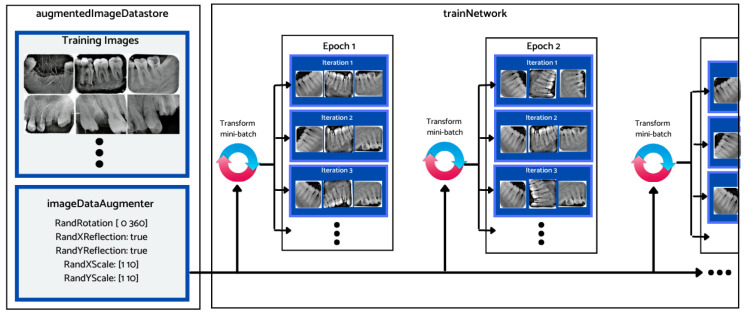
Data augmentation workflow.

Considering the relatively small size of the dataset, different data augmentation techniques were employed prior to training and evaluation of the different deep learning architectures’ performance. Data augmentation is a common preprocessing technique that is employed before feeding the data samples to the neural network for training. It entails creating artificial data from known data. This helps to mitigate the problem of overtraining and the adverse impact of class imbalance, leading to improved model accuracy. Figure 7 depicts how the augmented image datastore was used to transform the training data for each epoch. During this process, one randomly augmented version of each image was used during each training epoch.

**Figure 8:**
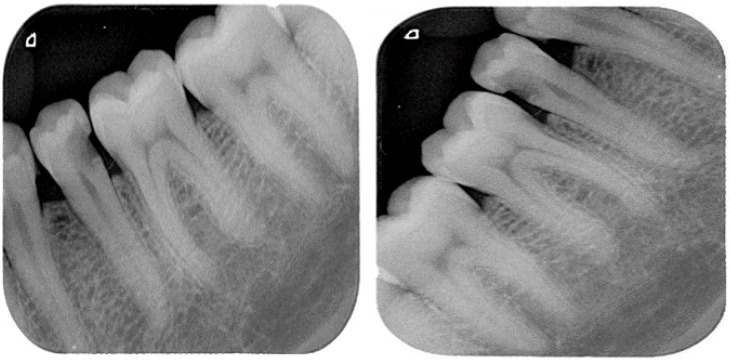
Original image and horizontally random reflected image.

The training dataset was augmented using an augmentation technique defined through suitable function classes, such as RandXReflection and RandYReflection, in Matlab. In addition to applying the data augmentation techniques, images input to Alexnet were resized and converted to color from grayscale. Using the data augmentation techniques, the number of training images was increased from 453 to 1359. Using RandYReflection, each image was reflected horizontally as shown in Figure 8.

**Figure 9:**
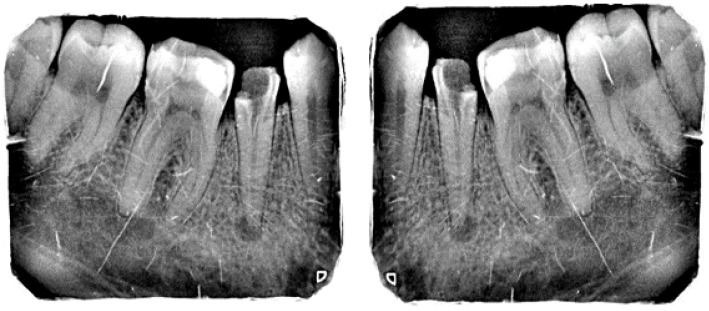
Original image and random vertically reflected image.

Figure 9 shows the RandXReflection-generated vertical reflection of each image. The images were augmented during training and were not saved in memory, and when the network parameters were trained, the augmented images were automatically discarded.

#### 3.2.2. Feature Extraction (Pre-Trained AlexNet)

A pre-trained AlexNet was used in the framework for feature extraction. This network comprises a large network structure with 60 million parameters and 650,000 neurons. Several improvements have been made to train the parameters [47]. One of the most significant improvements is the activation function. These functions provide nonlinearity within the neural network. One of the activation functions that helps in avoiding gradient vanishing problems is the rectified linear unit (ReLU) function. The calculation process of the ReLU function is

(1) ReLU(x)=max(x,o).

Furthermore, the ReLU function tends to converge faster than other activation functions in deep networks. A dropout layer is employed to avoid overfitting by forcing neurons to cooperate with others, resulting in improved generalization. The fully connected layers within the network are used for classification. The activation function utilized with the final layer was the softmax function, which constrains the output to a range of (0,1). Equation (2) below expresses the softmax function:

(2) $$softmax\left(x\right)=\frac{exp(x_{i})}{\sum_{j=1}^{n}exp\left(x_{j}\right)}.$$

The AlexNet model used in this study was made up of 25 layers, containing one input layer, five convolution layers, seven ReLU layers, two normalization layers, three max-pooling layers, and three fully connected layers.

#### 3.2.3. AlexNet Fine-Tuning Using Transfer Learning

The periapical radiographs dataset contained only a few hundred samples, which is insufficient to train a deep network of large volume such as AlexNet. Therefore, transfer learning was employed to make the network more suitable for feature selection and reduce the time required for classification. The entire structure of the network was divided into two parts: pretrained networks and transferred networks. The last three layers of the AlexNet were replaced: a fully connected layer with 1000 neurons to receive the extracted features and map them to output categories, another SoftMax layer, and a classification layer to output the classes; the rest of the model was preserved. The network parameters were already pretrained on ImageNet, making the extracted features effective for classification.

**Figure 10:**
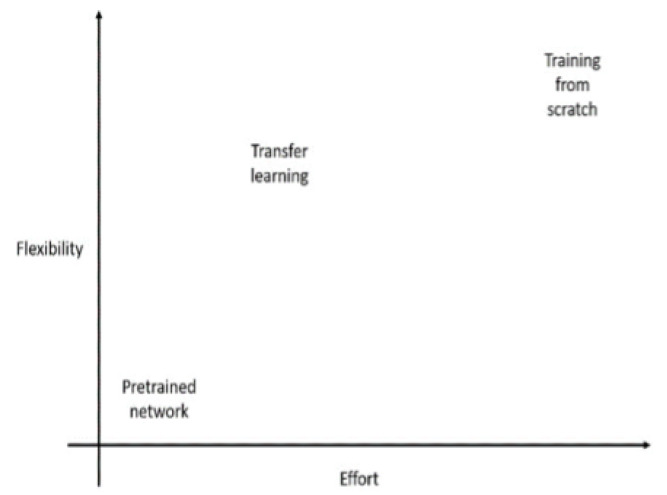
Effort vs. flexibility for transfer learning.

Transfer learning is a process that involves taking a pretrained network, modifying it, and retraining it on new data. This technique requires little data and computational time, as shown in Figure 10.

**Figure 11:**
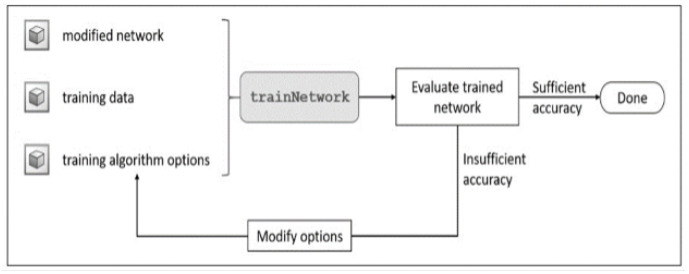
Workflow of the transfer learning approach.

Transfer learning is an effective and convenient method for training deep neural networks even when the system lacks sufficient labeled samples. Furthermore, transfer learning can be implemented using ordinary personal computers, making it more suitable for training networks with computational resource constraints. The workflow of transfer learning is shown in Figure 11.

**Figure 12:**
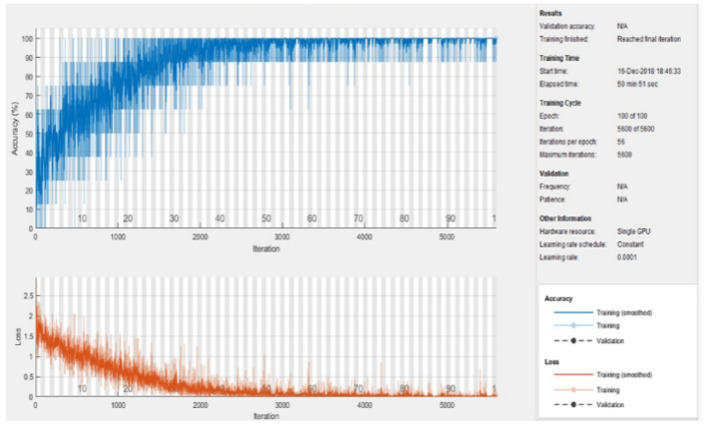
Training process of the model.

The learning rate was fixed at 0.0001 and the momentum value was kept the same as in the pretrained AlexNet, i.e., 0.9. The mini-batch size was set to 8 and the number of epochs was kept at 100. Using an NVIDIA graphics card (GeForce 930MX), the total training time was computed at around 50 min and 51 s, as depicted in Figure 12.

#### 3.2.4. Classification

Using the fine-tuned AlexNet, 4096 features were extracted from each periapical radiograph. The extracted features were then used to train two classifiers: support vector machine (SVM) and K-nearest neighbors (KNN). SVM allows for solving classification and regression-related problems by utilizing multi-dimensional hyperplanes to separate the data with more significant gaps between them, while KNN is a supervised data classifier based on lazy learning. It allows for storing data classes and classifying unseen data based on feature similarities from the known classes. Additionally, different functions can be used for feature comparisons, such as Manhattan, Euclidean, and Minkowski. Finally, the anonymous data are classified according to the nearest neighbor, with K being the number of neighbors.

#### 3.2.5. Performance Evaluation Metrics

The performance of the proposed approach was evaluated using accuracy for five classes by dividing the number of correct predictions by the total number of test X-rays. Equation (3) was used to evaluate accuracy [48]:

(3) $$Accuracy=\frac{TP+TN}{TP+TN+FP+FN}.$$

Accuracy is an important evaluation indicator in neural network models. To calculate the accuracy, the concepts of true positive (TP), true negative (TN), false positive (FP), and false negative (FN) are introduced [49].

#### 3.2.6. Error Analysis

In order to calculate the error between the predicted labels and the predicted value from the network, the Huber loss function [50] was used. The Adam optimizer was applied after the error was calculated to update the network biases and weights in each layer. The Hubert loss function leads to robust training and has proven to be effective in tracking outliers. It is defined as

(4) $$f_{huber}=\int_{\sigma }^{\frac{a^{2}}{2}}\left(\left|a\right|-\frac{1}{2}\right)\;a>\sigma ,a<\sigma ,$$

where σ is the threshold.

## 4. Experiments, Results, and Discussions

### 4.1. Settings

The proposed method was implemented using Matlab R2017a with an i5-4770 CPU and NVIDIA GeForce 930MX graphics card. The trained structure can run on any personal computer with Matlab. Additionally, 80% of the dataset was used for training, and the rest was used for testing. The details of the dataset after augmentation are listed in Table 3.

### 4.2. Experiments

#### 4.2.1. First Set of Experiments

**Figure 13:**
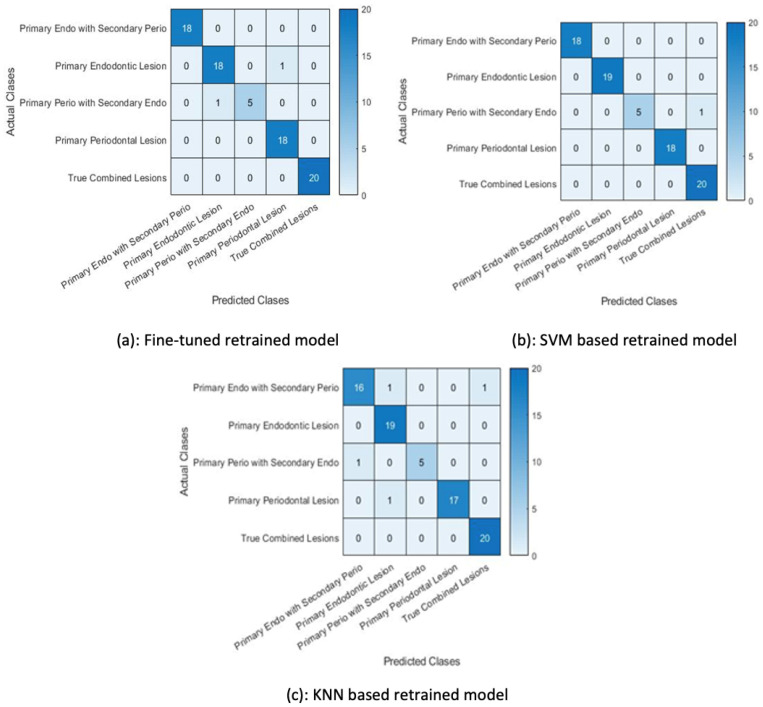
Confusion matrices for first set of experiments.

The features were extracted using the pretrained Alexnet and the extracted features were used to train SVM and KNN classifiers. Feature extraction using the pretrained deep convolution neural network was effortless and quick. A suitable function from the statistic and machine learning toolbox was used to train the multi-class SVM. Similarly, an appropriate function from the statistic and machine learning toolbox was used to train the KNN on the features extracted from the training data. The confusion matrix function was used to show the confusion matrix for the predictions of both classifiers. The SVM classifier performed better than the KNN for this set of experiments. The classification accuracy achieved by the two classifiers is shown in Figure 13.

#### 4.2.2. Second Set of Experiments

In the second set of experiments, the pretrained Alexnet network was fine-tuned with the augmented data. This retrained network was then used to classify the test examples using the softmax function and the same retrained model was used for feature extraction. The ReLU layer (21st layer) of the retrained model was used to output the extracted features. The SVM and KNN were then retrained on these extracted features by changing the activation function.

**Figure 14:**
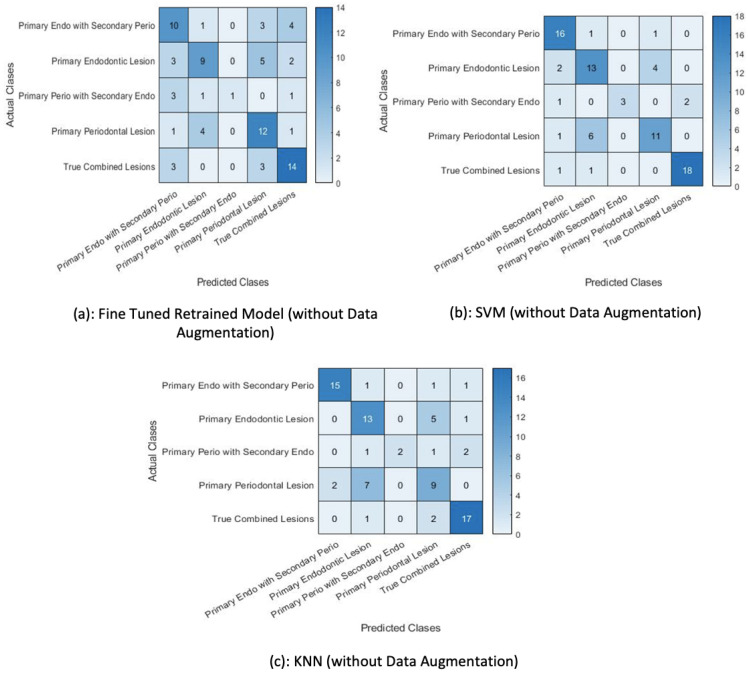
Results for the second set of experiments.

The classification accuracy in Figure 14 shows that the SVM trained on the features extracted by the retrained network fine-tuned on augmented data performed better than the other two classifiers, i.e., the KNN classifier and the retrained (softmax classifier).

The classification of test data by the retrained model misclassified two examples. It wrongly predicted a primary endo lesion instead of a primary perio with secondary endo lesion and a primary periodontal lesion for a primary endo lesion, as shown in Figure 14a. The next figure shows the confusion matrix of the SVM trained on features extracted by the retrained model, demonstrating that the trained SVM is unable to classify one test example. The KNN classifier trained on the features extracted by the retrained model wrongly classified four test examples, as shown in Figure 14c.

### 4.3. Fine-Tuned AlexNet without Data Augmentation

The pretrained network (AlexNet) was fine-tuned using the training data without augmentation and its performance was compared against the AlexNet trained with data augmentation in order to establish the efficacy of the former in classifying test images using the softmax function. The training set was kept at 453 training examples and the network’s performance versus the amount of data provided for training was computed. It can be observed that the performance of the pretrained Alexnet deteriorated without the data augmentation approach.

**Figure 15:**
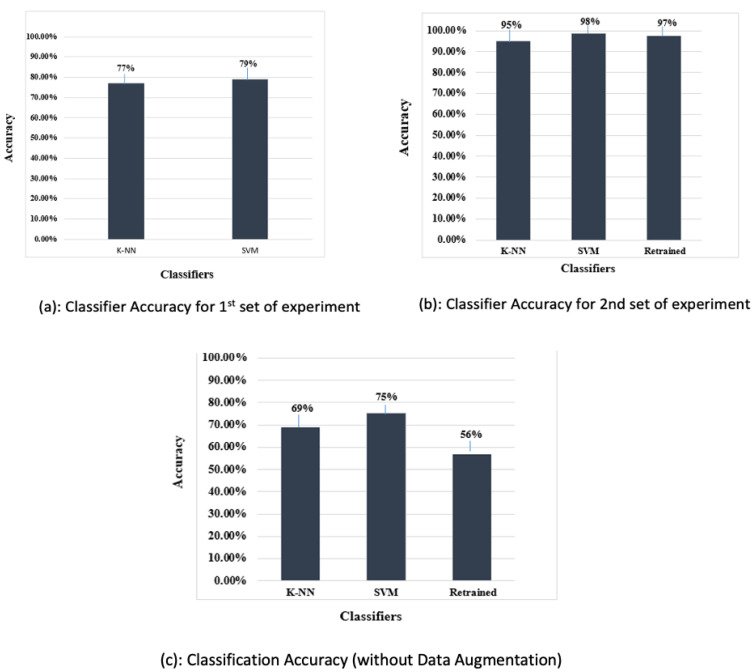
Percentage accuracy of all classifiers.

A total of 81 test examples were passed through the retrained model for classification, and 35 examples were misclassified by the retrained network. The same test data were presented to the SVM and KNN classifiers trained on this retrained AlexNet model. The results show that lower accuracy was achieved by the classifiers when data augmentation was not performed. The classification accuracy depicted in Table 4 indicates that the SVM trained on features extracted by the retrained model fine-tuned with the augmented data performed better than the other classifiers. Additionally, Table 5 shows the precision, recall, and f1-score achieved using the proposed classifier. Figure 15 shows the accuracy of classifiers for different scenarios.

The IoDT has great potential in opening up new horizons in the dental field. The proposed work incorporates a IoT-based dental film to capture and transfer X-rays automatically to ensure that radiographs can be analyzed effectively. The fine-tuned AlexNet was trained using periapical radiographs belonging to different classes. The minimum batch size was set to 8 to avoid low memory issues. The minimum learning rate was set to 0.0001 to mimic a slow learning pace, allowing newly added layers to catch up to existing layers. To show the efficacy of the proposed approach, it was compared with several state-of-the-art methods, as shown in Table 4. The results show that the SVM trained on features extracted by the retrained model fine-tuned with the augmented data performs better compared to other classifiers. Furthermore, the loss ratio is small even when using a smaller dataset. These good results are achieved through transfer learning. Testing was carried out using 81 images, and confusion matrices were produced for the different sets of experiments.

Furthermore, other advanced deep learning-based methods can be tried for periapical radiographs. More robust data augmentation could be applied to further enhance the dataset to achieve improved results. The model can perform multi-class classification on periapical radiographs. However, other intraoral radiographs could be used to further validate the efficacy of the proposed model. The future management of dental caries will be based on three factors: early disease detection, assessment of risks, and prevention. The aim of this study is to provide affordable alternatives for patients that can be used effectively at the community level.

### 4.4. Performance Analysis Using State-of-the-Art Approaches

To corroborate the efficacy of the proposed approach, a performance appraisal was carried out with other state-of-the-art approaches; the results are provided in Table 6. The proposed method achieves an accuracy of 98% using data augmentation and 75% without data augmentation. These results confirm the superior performance of the proposed approach over recent approaches used for diagnosis of teeth lesions.

### 4.5. Strengths and Limitations of the Proposed Approach

The proposed approach has several strengths and limitations that are worth considering when evaluating the real-time deployment of the proposed system.

The proposed approach utilizes deep learning-based techniques such as data augmentation and transfer learning, which are more effective in detecting complex dental lesions. Furthermore, more accurate and reliable data collection can be ensured by integrating IoT-enabled devices. The need for expert involvement is significantly reduced, making the system more accessible to patients.

However, there are certain limitations to the proposed approach. First, it requires access to periapical radiographs, which might not be readily available due to privacy concerns. Second, the accuracy of the proposed system relies on the accuracy of the IoT-enabled device used for data collection. This can be influenced by different factors, such as device calibration and lighting conditions. Third, the proposed model may not be as effective in detecting other types of periapical lesions, as the model was specifically trained on periapical radiographs. Lastly, there may be scalability issues in resource-constrained environments, which may have a negative impact on the performance of the proposed model.

Keeping in view these limitations, several factors could influence the real-world deployment of the proposed system. These include the availability of periapical radiographs in dental clinics where the system is to be deployed, the cost and accessibility of IoT-enabled devices, the availability of computational resources, and regulatory ethical considerations related to the use of patient data for model training and testing.

## 5. Conclusions and Future Work

This work has presented an endo perio lesion detection system using the transfer learning and data augmentation approach involving multiple processes without involving an expert dentist. The proposed technique utilizes data acquisition using an IoT-enabled mouth guard; the data are preprocessed and used to extract suitable features with a pretrained AlexNet CNN through an augmentation approach. We additionally investigated the utilization of transfer learning in dental lesion recognition based on intraoral radiographs. CNNS trained using transfer learning require a lower amount of data compared to CNNs trained from scratch. The proposed model utilizes a pretrained AlexNet, and fine-tuned classification was performed using the SVM classifier to recognize five classes using 453 images, then tested on 81 images. The performance of the proposed method was evaluated against other conventional classifiers and achieved 98% accuracy, demonstrating the effectiveness of our approach. The proposed model can be applied in daily clinical diagnosis to help dentists make decisions to improve patient care.

Even though the model provides encouraging results, it could be further improved by utilizing more robust data augmentation techniques and more the resource-hungry deep learning methods at the cost of computing resources. Additionally, the use of dental cone beam computed tomography images, bitewing radiographs, and panoramic X-ray images could be considered in future work. Moreover, the design and development of a dashboard in the form of a smartphone application to allow the user to obtain intelligent feedback and track dental health issues could be undertaken as well.

## Figures and Tables

**Table 1:** Review of the research works discussed in this section.

Reference	Methodology	Task	Journal
Kühnisch et al. [19]	CNN, data augmentation, transfer learning	Classification	Journal of Dental Research
Duong et al. [20]	International Caries Detection and Assessment System (ICDAS) II, SVM	Classification	Health Informatics Journal
Schwendicke & Paris [39]	Resnet18, Resnext50	Classification	Journal of Dentistry
Gawad et al. [38]	2-D Hilbert Transform, Inspection Algorithm	Classification	Lasers in Dental Science
Geetha et al. [33]	ANN, 10-fold cross-validation	Classification	Health Information Science and Systems
Patil et al. [30]	MPCA based Feature Extraction, NN Classifier	Classification	Health Informatics Journal
Casalengo et al. [32]	CNN trained on a semantic segmentation task	Classification	Journal of Dental Research
Salehi et al. [35]	CNN with Optical Coherence Tomography (OCT)	Classification	Lasers in Dentistry
Kim et al. [40]	High Frequency Ultrasound Imaging (HFUS)	Classification	Journal of Dental Research
Lee et al. [2]	CNN	Classification	Journal of Dentistry
Srivastava et al. [41]	FCNN (Deep Fully Connected, Convolutional Neural Network)	Segmentation	NIPS 2017 workshop on Machine Learning for Health
Prajapati & Nagaraj [22]	CNN	Classification	5th International Symposium on Computational and Business Intelligence

**Table 2:** Periapical radiographs dataset.

Lesion Type	Images
Primary Endo with Secondary Perio	122
Primary Endodontic Lesion	124
Primary Perio with Secondary Endo	39
Primary Periodontal Lesion	118
True Combined Lesions	131
Total radiographs	534

**Table 3:** Training and testing data et of periapical X-rays.

Lesion Type	Training Data	Test Data
Primary Endo with Secondary Perio	104	18
Primary Endodontic Lesion	105	19
Primary Perio with Secondary Endo	33	06
Primary Periodontal Lesion	100	18
True Combined Lesions	111	20
Total radiographs	453	81
Total X-rays after data augmentation	1359	

**Table 4:** Accuracy Results for Classifiers.

Classifier	Accuracy (%)	
	With Data Augmentation	Without Data Augmentation
SVM	98	75
KNN	97	56
Retrained	95	69

**Table 5:** Precision, recall, and F1 score results for classifiers.

	Precision	Recall	F1 Score
Primary Endo with Secondary Perio	0.761	0.888	0.819
Primary Endodontic Lesion	0.619	0.684	0.649
Primary Perio with secondary Endo	1.0	0.5	0.666
Primary Periodontal Lesion	0.687	0.611	0.646
Trued Combined Lesions	0.9	0.9	0.9
Macro avg	0.793	0.716	0.736
Weighted avg	0.757	0.751	0.748

**Table 6:** Performance comparison with state-of-the art models.

Author, Year	Methodology	Accuracy (%)
Kühnisch et al., 2021 [19]	CNN, Image augmentation, Transfer Learning	90.6%
Duong et al., 2021 [20]	ICDAS II, SVM	92.37%
Schwendicke & Paris, 2020 [39]	Resnet18, Resnext50	94%
Geetha et al., 2020 [33]	ANN, 10-fold cross-validation	97.1%
Proposed approach	CNN, transfer learning, deep training	98%

## Data Availability

Not applicable.
